# Low Cognitive Load and Reduced Arousal Impede Practice Effects on Executive Functioning, Metacognitive Confidence and Decision Making

**DOI:** 10.1371/journal.pone.0115689

**Published:** 2014-12-30

**Authors:** Simon A. Jackson, Sabina Kleitman, Eugene Aidman

**Affiliations:** 1 School of Psychology, University of Sydney, Sydney, Australia; 2 Land Division, Defence Science and Technology Organisation, Adelaide, South Australia, Australia; University of Tuebingen Medical School, Germany

## Abstract

The present study investigated the effects of low cognitive workload and the absence of arousal induced via external physical stimulation (motion) on practice-related improvements in executive (inhibitory) control, short-term memory, metacognitive monitoring and decision making. A total of 70 office workers performed low and moderately engaging passenger tasks in two successive 20-minute simulated drives and repeated a battery of decision making and inhibitory control tests three times – before, between and after these drives. For half the participants, visual simulation was synchronised with (moderately arousing) motion generated through LAnd Motion Platform, with vibration levels corresponding to a well-maintained unsealed road. The other half performed the same simulated drive without motion. Participants’ performance significantly improved over the three test blocks, which is indicative of typical practice effects. The magnitude of these improvements was the highest when both motion and moderate cognitive load were present. The same effects declined either in the absence of motion (low arousal) or following a low cognitive workload task, thus suggesting two distinct pathways through which practice-related improvements in cognitive performance may be hampered. Practice, however, degraded certain aspects of metacognitive performance, as participants became less likely to detect incorrect decisions in the decision-making test with each subsequent test block. Implications include consideration of low cognitive load and arousal as factors responsible for performance decline and targets for the development of interventions/strategies in low load/arousal conditions such as autonomous vehicle operations and highway driving.

## Introduction

The ability to learn and maintain optimal operational performance is critical in many domains. In a general sense, this can occur when individuals are fully engaged with the task at hand and have access to the necessary resources [1]. Under the umbrella of cognitive fatigue, much research has explored the ways in which deviations from this desired state occur. Following the work of Yerkes & Dodson [2], deviations from optimal performance are thought to result from workload and arousal levels that are too great or too low. Considerable focus has been on the overload of cognitive resources due to sustaining high workload (see [1], [3]). In these cases, more information is received than can be processed. This type of fatigue typically manifests as a performance decline accompanied by subjective reports of high mental workload (e.g., effort and frustration) as measured by scales like the NASA Task Load indeX (NASA-TLX).

In contrast, there is a growing interest in task disengagement as a result of low workload, thought to yield similar performance declines without reports of high mental effort. In low workload conditions, individuals can process more information than they receive and may lose interest in the task at hand due to low arousal levels. This leads to the mind slowing down and generating distractions. Reaction speeds on monotonous vigilance tasks consistently slow after sustained performance (e.g. [4]). Participants also report an increase in task-irrelevant activity such as mental singing and counting to cope with these tasks (e.g. [3]). Hart [5], for example, found that unmanned vehicle operators, prompted to re-plan command strategies every 10, 20 or 30 minutes amid autonomous surveillance, created extra unnecessary work. Hart concluded that the operators craved at least moderate workload to the degree that they would self-impose it unnecessarily.

Research on low workload has developed along two lines: boredom and passive fatigue. Boredom, mainly studied in education and organisational settings, is described as a psychological state characterised by low arousal and subjective disengagement [6]. Boredom has been shown to reduce attendance rates, and hence learning outcomes, in academic contexts [7] and decision-making performance in operational and organisational contexts [8], [9]. In the same vein, passive fatigue, studied in human factor/ergonomic settings, is thought to occur when there is low cognitive workload and a lack of direct control over the task at hand [10]. This line of research has shown that passive fatigue leads to overall performance decrements in operator conditions. For example, increased vehicle automation is associated with reduced driver vigilance indicated via slower time to brake or steer away in response to an emergency event [1], [11]. The binding feature across the boredom and passive fatigue lines of research is that low levels of cognitive workload and environmental arousal are considered to cause deterioration in performance.

In either field, however, the effects of these variables on practice-related improvements have not been studied. The focus of the present experiment was to investigate how low levels of cognitive workload and arousal induced by external physical stimulation (a typical source of environmental arousal in operational contexts such as driving) may contribute to non-optimal levels of practice-related learning.

### Cognitive load and arousal

To investigate cognitive load in the present study, participants were instructed to sustain attention as passengers during two 20 minute simulated drives. They were told that they would be asked questions about what they saw. The simulated road was in the country and required little cognitive resources: three to four gentle turns, a few sections of trees, a barn, four to five speed limit signs and no other traffic. Cognitive load was manipulated within subjects such that one drive involved nothing else, while the other included a secondary cognitive task (described in method). With no other task to complete, the amount of information to be processed was minimal and this drive constituted the low-load condition. Engagement with the secondary cognitive task placed additional cognitive load on the participants and this drive constituted the moderate-load condition. Based on Scerbo’s [4] findings that vigilance performance remained low despite task variations, low load might have a sustained impact on performance despite a return to moderate load. To investigate this, the order in which participants experienced the low and moderate-load drives was counterbalanced. A country road, rather than frequently studied monotonous highways, was used to selectively target cognitive load. The good conditions and regular geometry of monotonous highways have been implicated as a cause of decreasing arousal in addition to low cognitive load [12]–[14].

Environmental arousal was manipulated between subjects by randomly allocating participants to either a motion or no-motion version of the same simulated drive. Vibration levels under the motion condition corresponded to a well-maintained unsealed road, which is considered to be moderately arousing. Thorough experimentation utilising motion beyond this level had not been conducted with the current simulator apparatus. Increasing motion beyond this level was therefore considered unsafe to participants and potentially hazardous to the integrity of the experiment. Higher motion levels were therefore not used for safety reasons and to ensure that over-arousal was not a factor.

For all participants, the two drives were intermixed between three administrations of the same test battery assessing key decision making factors: executive (inhibitory) control, short-term memory, metacognitive confidence and decision-making competence. The goal was to systematically assess convergent changes in these decision-making factors as a result of practice and the influence of low cognitive load and a lack of arousal.

### Practice Effects

Practice Effects (PEs) refer to improvement in performance associated with repeated administrations of the same or similar test items [15], [16]. They reflect declarative learning of repeated items and procedural learning of test relevant strategies independent of item content [17], [18]. Procedural learning through practice is of crucial importance in real-world contexts that rarely repeat but frequently require similar strategies.

PEs are naturally embedded in repeated-measures experimental designs. Until recently, PEs were treated as a psychometric nuisance and typical procedures attempted to control for them by introducing multiple baselines [15], [19]. Recent evidence, however, suggests that PEs are an indication of cognitive ability in their own right (e.g. [18]). That is, cognitive decline can be inferred when typical PEs are not observed. Given the evidence described above, we hypothesised that low cognitive load and low arousal would result in a reduction of PEs on all tests assessing factors involved in competent decision making.

### Decision making

Decision making involves forming judgements about the world and making choices to achieve one’s goals [20]–[22]. Judgements vary in their accuracy and can be generated via two processes: Type 1 processes are effortless, automatic and intuitive; Type 2 processes require effort and deliberation [22]. When judgements about the task at hand are accurate, the most competent thing to do is act confidently and decisively to achieve ones goal. When accurate judgements about the problem cannot be formed, however, the most competent thing to do is address various ancillary goals, such as seeking more information. A competent military commander, for example, will take a route they accurately judge to be safe (the primary goal is to deliver the team to the destination in the safest and quickest way possible) but radio higher command otherwise (addressing the ancillary goal of obtaining more information in support of achieving the primary goal). Less competent commanders might take dangerous routes, incorrectly concluding that they are safe, or waste time by requesting further information from higher command despite having already identified the safest paths. The present study investigated how a reduction in cognitive workload (through secondary task manipulation) and arousal (induced through motion manipulation) modulates practice effects on a range of variables involved in the decision-making process.

### Inhibitory control

Decisions are made after reflecting on their consequences [23]. As such, decision making relies on the executive function of inhibitory control: top-down mental processes that maintain and direct attention in the face of automatic thinking [24]. The impact of inhibitory control on decision making is two-fold: first, it involves suppressing dominant, automatic or pre-potent Type 1 responses and overriding them with more deliberate Type 2 processes. Disturbances in this mechanism may relate to impulsivity and reckless decision styles. For example, a novice commander might notice evidence of a large explosion on one route and intuitively judge it to be less safe than another. A decision derived from these first impressions alone would be incomplete and lacking a more deliberate Type 2 analysis that involves multi-criterion reasoning. As well as ensuring the suppression of Type 1 responses, inhibitory control is responsible for maintaining Type 2 processes and resisting distraction, as it underpins the capacity to concentrate on the task at hand and to filter out unwanted or irrelevant input.

In the present study, the colour naming Stroop [25] test was included in the repeated test battery as a standard measure of inhibitory control. Repeated performance on this test tends to produce PEs in the form of faster response times and fewer errors. We hypothesized that lower levels of cognitive workload or arousal would decrease the magnitude of these PEs.

### Cognitive abilities and short-term memory

Type 2 processes that generate accurate judgements include “higher level” cognitive abilities. For example, individuals with greater capacity for reasoning, short-term memory or long-term storage and retrieval are able to accurately process more information than less able individuals [26]. PEs are consistently observed on typical cognitive ability tests as improvements in test scores. For example, people develop superior strategies that aid their memory with repeated practice on tests of short-term memory. The present study used a Medical Decision-Making Test (MDMT; described in method) that requires participants to memorize a difficult list of symptoms in order to diagnose patients, therefore depending on their short-term memory [21]. In addition to short-term memory performance, the MDMT is the only psychometrically validated test available that also captures metacognitive confidence and the decision variables described below. Employing more tests and capturing a broader array of cognitive abilities was not possible given our experimental timeframe. Our hypothesis was that participants would score higher with each administration of this short-term memory test (PE), but that lower levels of cognitive workload or arousal would hamper these improvements.

### Metacognitive monitoring

The monitoring of Type 2 processes tends to be studied under the domain of metacognition, defined as “thinking about thinking” or “knowing about knowing” (e.g. [27], [28]). Good metacognitive abilities–here referred to as the ability to monitor judgement accuracy–are crucial for competent decisions to be made. Common to different theories, metacognition involves various sub-components and skills (e.g. [29]). A complete analysis of these, however, is outside the scope of the present research (see [29], [30] for reviews). Our focus will thus be on the ability to monitor and assess judgement accuracy only.

Metacognitive monitoring gives rise to on-task experiences of confidence and post-task evaluations such as retrospective appraisals of cognitive demand or performance. On-task confidence is an experience of accuracy that directs present decisions [21], [31] while post-task evaluations guide future performance. As such, individuals tend to act decisively to address their goals when they are confident, but take action to address ancillary goals when they lack confidence in their judgements [21], [22], [31]. Similarly, individuals may be inclined to change their approach in the future when post-task performance evaluations are low but not when they are high. In this study, participants were required to indicate their on-task confidence in each MDMT diagnosis (from 0% no confidence at all to 100% completely confident), and indicate how successful they were at completing the MDMT immediately after each administration as assessed by the NASA-TLX performance scale. The NASA-TLX was used as the subjective post-task assessment of workload in the present study as it has been shown to correlate stronger with objective task performance than alternative measures [32].

Convergent changes in PEs have not been thoroughly investigated for both on- and post-task monitoring variables. Paese & Sniezek [33] hypothesised that on-task confidence is likely to increase with practice as people believe that practice will benefit their ability to perform the task at hand. Indeed, they found that participants became more confident in judging the ability of baseball players with practice despite no increase in the accuracy of their judgements. It is important to distinguish these variables from monitoring variables captured post-learning but prior to evaluative tests. For example, Judgements-of-Learning (JOL), which are captured post learning but prior to tests of that learning tend to remain stable across practice periods despite improvements in the accuracy in the subsequent tests [34], [35]. Variables such as JOLs, however, are theoretically distinct from post-task performance evaluations captured by the NASA-TLX, as they capture a different aspect of the monitoring process. Although important, our focus in the present study will be the on- and post-evaluative task monitoring variables described thus far. Given Paese & Sniezek’s [33] findings, we therefore hypothesised that both on-task confidence and post-task performance evaluations would increase with practice, but that lower levels of cognitive workload or arousal would impede these changes.

### Decision competence

The factors involved in the control of cognition, and formation and checking of judgements are what underpin various decision behaviours. In operational contexts such as driving or military command and control, profiling consistent patterns of decision behaviour is best achieved with Decision Pattern Analysis (DPA; [21]). Using the MDMT, the present study will track changes in DPA variables as additional markers to investigate their convergent response to PEs and low cognitive load and low arousal.

**Figure 1:**
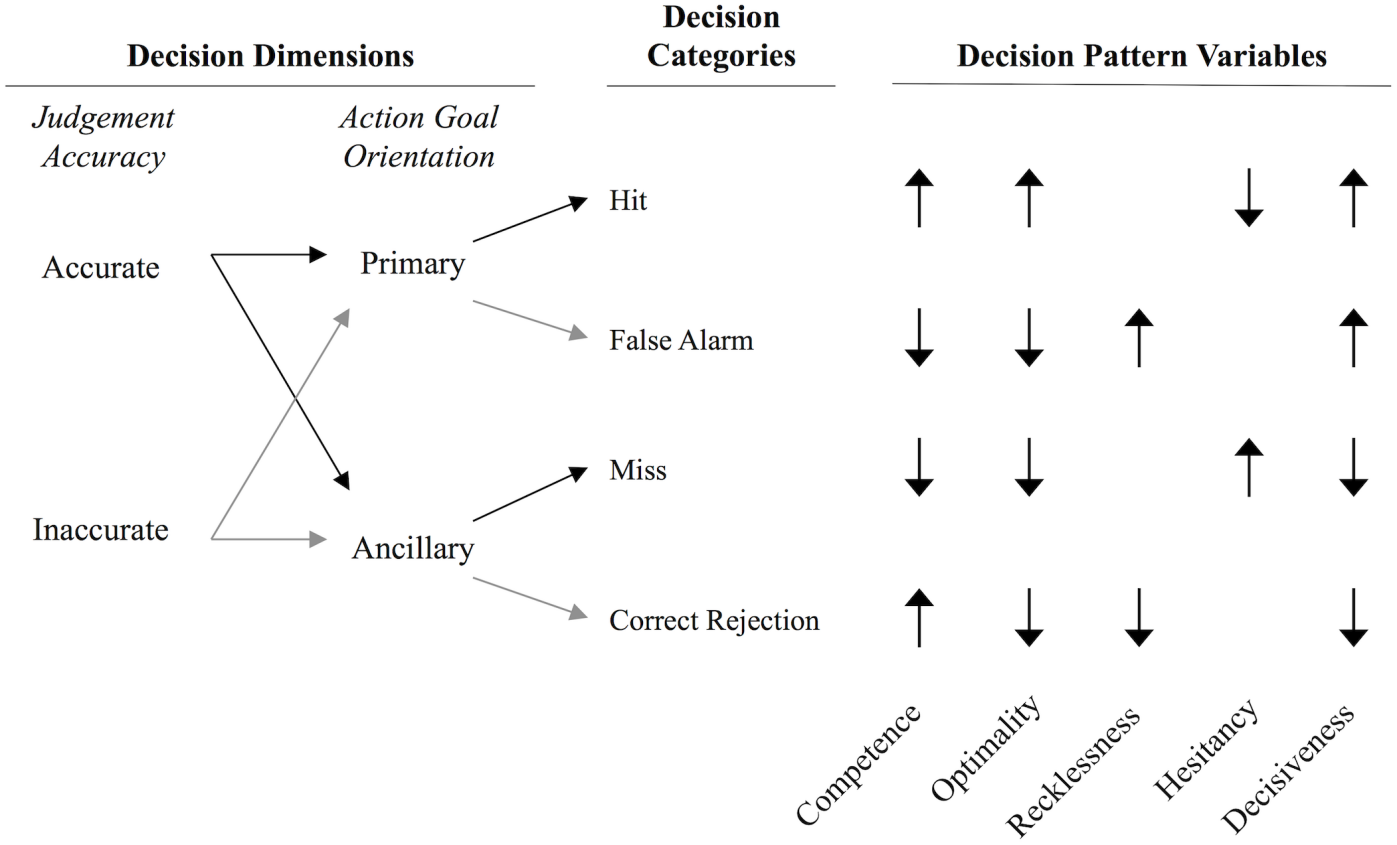
Decision dimensions, categories and pattern variables of Decision Pattern Analysis. Black lines from Judgement Accuracy to Decision Categories represent decisions made following accurate judgements; Grey lines represent decisions made following inaccurate judgements. Under Decision Pattern Variables, upward pointing arrows represent decision categories that increase the score on that decision pattern; downward pointing arrows represent decision categories that decrease the decision pattern score.

DPA classifies decisions on the basis of two dimensions: judgement accuracy and goal orientation. Already discussed, a decision based on accurate judgements and oriented toward addressing one’s primary goal is competent. Also competent is a decision addressing ancillary goals when inaccurate judgements are held. These cases are categorised as *hits* and *correct rejections*. Incompetent decisions, however, are those based on inaccurate judgements but still oriented towards the primary goal (categorised as *false alarms*), or decisions addressing ancillary goals despite already holding accurate information (*misses*). These cases are represented in Fig. 1.

DPA uses multiple decisions categorised as hits, correct rejections, false alarms and misses to describe individuals’ consistent patterns of decision behaviour. The patterns are captured with the following five variables, which have received sound empirical support for their reliability and validity [21], [22], and are depicted in Fig. 1. *Competence* is a pattern of decision behaviour characterised by acting appropriately given the accuracy of one’s judgements. It is thus calculated as the number of *hits* and *correct rejections* given as a percentage of all decisions made. High scores reflect competent decision making and low scores incompetent (where more *false alarms* and *misses* are being made). A competent commander, for example, takes safe routes immediately but radios higher command when dangerous routes are encountered.

*Optimality* is a special case of competence characterised by the ability to make accurate judgements and act on them. It is thus computed as the number of *hits* given as a percentage of all decisions made. An optimal commander, for example, can make accurate judgements about the environment and act immediately on them.

*Recklessness* is an incompetent pattern of decision behaviour characterised by the inappropriate pursuit of primary goals. It is thus calculated as the number of *false alarms* given as a percentage of all inaccurate judgements. A reckless commander would tend to take dangerous routes despite incorrectly judging them to be safe.

*Hesitancy*, another type of incompetent behaviour, is characterised by the unnecessary pursuit of ancillary goals. It is thus computed as the number of *misses* given as a percentage of all accurate judgements. A hesitant commander, for example, tends to unnecessarily radio higher command for advice despite having made accurate judgements about the environment.

*Decisiveness* simply captures the tendency to pursue primary goals. It is thus computed as the number of decisions to address the primary-goal (*hits* and *false alarms*) as a percentage of all decisions. A decisive commander, for example, tends to immediately take the route s/he identifies as being safest (regardless of whether they are correct or not).

To date, no research has investigated the effects of either practice or low cognitive load and arousal on these variables. However, given the positive nature of PE, we hypothesised that competence and optimality would increase, and recklessness and hesitancy would decline. We also hypothesised that as confidence is expected to increase, so will overall decisiveness. As with the other variables, we hypothesised that lower levels of cognitive load and arousal may independently impede these positive changes.

### Aims and hypotheses

The primary aim of the present study was to simultaneously assess changes in practice-related improvements, or lack of them, in inhibitory control, short-term memory, metacognitive monitoring, and patterns of behavioural decision competence/incompetence under conditions of low cognitive load and arousal. To achieve this, pre to post-changes in these variables were tracked over three testing blocks (providing practice) interspersed by two 20 minute simulated drives. One drive required sustained attention on the road only (low load), while the other required a cognitive task to be completed as well (moderate load). All participants experienced both drives, but the order in which they were experienced was manipulated. Participants were additionally allocated to experience both drives in a motion or no-motion condition. To our knowledge, the simultaneous effects of low cognitive workload and a lack of arousal induced via motion on a range of decision-making factors and behaviours have not been investigated. Following is a summary of our main effect hypotheses. Given the novelty of our research, interactions will also be examined but no directional hypotheses about them will be made.

*H1. Practice effects (PE) will be positive*. Participants will improve on all variables in a linear fashion with each additional test block. This will be indicated by:

1.1.Increases in judgement accuracy, on-task confidence, competence, optimality and decisiveness within the MDMT, and post-task performance evaluations in relation to this test (captured by NASA-TLX).

1.2.Decreases in reaction time and errors on the inhibitory control Stroop task, recklessness and hesitancy in the MDMT, and post-task evaluations of temporal, physical and mental demand, as well as effort and frustration in relation to this test (as measured by NASA-TLX).

*H2. Low cognitive load and arousal will impede PE.* The positive PEs described in H1 will be weaker under the low-load and no-motion conditions, i.e.:

1.3.following the low-load compared to moderate-load drive.

1.4.for participants receiving no motion compared to those with motion.

*H3. Low cognitive load will have a sustained effect* such that the PEs described in H1 will be weaker overall for participants who start with the low-load drive (and do the moderate-load drive second) than participants who start with the moderate-load drive first (and have low-load second).

## Method

### Participants

A total of 70 participants (7 female, *M*_age_ = 37.71 years, age range: 19–60 years), recruited via email invitations to Defence Science and Technology Organisation (DSTO) personnel, volunteered to participate. Participants were briefed and provided written consent prior to the experiment beginning, and they were debriefed immediately after the experiment concluded. Power analysis conducted with G*power [36] indicated that a sample size of 60 was sufficient for detecting small to moderate effects given the present design and assuming a correlation of.50 between repeated measurements across the three test blocks.

### Ethics Statement

Ethics approval (LD 09-13) has been granted by the Defence Science and Technology Organisation (DSTO) Low Risk Human Research Ethics Review Panel.

### Materials

#### Control variables

1. *Short Motion sickness questionnaire* [*37*] *.* This questionnaire assesses susceptibility to motion sickness. For example, participants rate how often they felt sick on cars, buses and trains as a child and in the last 12 years. Participants reporting mild susceptibility to motion sickness were closely monitored or excluded if reporting considerable susceptibility.
2. *Driving and Military Experience Questionnaire.* This questionnaire assesses demographic and physical variables–Age, Gender, dominant hand, eyesight and hearing quality–as well as driving and military experience, including experience with simulated environments such as those used in the present experiment.
3. *Self-control Scale* [*38*] *.* This self-report scale assesses the ability to interrupt and override automatic and undesirable behaviours. Participants rate to what degree each of 36 statements such as “I blurt out whatever is on my mind” (reverse scored), reflect how they typically are from 1 = Not at all to 5 = Very much. This was collected as a control variable that may account for baseline difference in inhibitory control on the Stroop test.

No participants were excluded on the basis of the above control variables.

1. *Attention switching tasks.* A total of ten attention-switching tasks were compiled in a paper booklet as filler tasks (see Procedure). In each task, participants are given a key of four symbols and their associated characters. Participants must then write down the characters that match a series of symbols as quickly as they can. Each task has a compatible version followed by an incompatible version. For example, the compass substitution test key requires the matching of four arrows to the letters N, S, E and W (for North, South, East and West). The compatible version has N for the upward arrow, E for the rightward arrow, etc. The incompatible version has N for the downward arrow, E for the leftward arrow, etc. This test was used as a time-filler for participants who finished responding earlier than others.

#### Repeated test battery

The following tests were administered at each testing block in the order in which they appear below. Participants took no more than 20 minutes to complete each block.

1. *Medical Decision-Making Test* [*21*] *.* In this test participants adopt the role of a specialist in fictitious fatal viruses to diagnose and treat infected patients. Participants have three minutes to memorise how nine symptoms (e.g., vomiting) can be used to make four possible diagnoses. After this, participants must diagnose 16 patients, each with a different combination of symptoms, with one of the four diagnoses. Participants indicate their confidence in each diagnosis (from 0% to 100% in 10% increments) and decide to treat each patient with an antivirus matching their diagnosis in an attempt to save their lives (primary goal) or request a blood test to obtain an accurate diagnosis (ancillary goal). Participants are told that administering a correct antivirus will save the patient (*hit*), but incorrect antiviruses will kill the patient (*false alarm*). The blood test will make a certain diagnosis, but patients only have a 50% chance to survive waiting for the results (*correct rejection* if incorrect diagnosis; *miss* if correct diagnosis). As described in the introduction, the five decision patterns (competence, optimality, recklessness, hesitancy and decisiveness) are computed at the end of the test based on the frequency of decisions classified as hits, correct rejections, false alarms and misses.

In the present study, two symptom lists were presented in a counterbalanced fashion for all participants across the three testing blocks. Over the three testing blocks, for example, a single participant might have had symptom list A in Block 1, followed by list B in Block 2, and list A again in Block 3. This was done to investigate PEs as a result of procedural learning of test strategies rather than declarative learning of repeated test content.

1. *NASA-TLX* [*39*] *.* The NASA-TLX assesses post-task evaluations of workload on six dimensions: Mental, physical and temporal demand, as well as performance, effort and frustration. It was administered immediately following, and in relation to, the Medical Decision-Making Test (MDMT) above. For example, after completing the MDMT, participants were asked how mentally demanding they found it? How successful they were in accomplishing it (performance)? Each workload dimension is measured with a single item rated on a scale from 1 being low workload/high performance and 21 being high workload/low performance.
2. *Stroop colour naming test* [*25*]. The Stroop colour naming test is the gold standard test of inhibitory control [40]. For each item in this test, participant must respond with the colour of a word that is presented as quickly as possible. Words can be coloured red, blue, green and yellow. The words themselves can be “RED”, “BLUE”, “GREEN”, “YELLOW” or “XXXXXX”. The word itself (not the colour) generates a prepotent Type 1 judgement, which may conflict with the colour. Trials can be classed as neutral when the word is “XXXXXX”, congruent when the word matches its colour, and incongruent when the word does not match its colour. Performance on each trial type is measured by mean response times (after excluding anticipatory responses <150 ms, lapses >2500 ms and errors) and the frequency of errors (incorrect colour reported). Greater inhibitory control is best indexed by faster reaction times and fewer errors on incongruent trials.

#### Secondary cognitive workload task

1. *Letter swaps test* [*41*] *.* In this test, participants are presented with a set of three letters (e.g., J–K–L) and instructed to mentally swap the position of two (e.g., swap 1 and 2; answer = K–L–J). They may be presented with many such instructions and must indicate the final order of the letter string. This was used as the moderate-load manipulation during one of the drives as it requires sustained working memory and attention. The complexity, and hence load of the task, can be manipulated by increasing the number of swap instructions. Stankov [41] found that participants were over 90% accurate for one to three instructions, and 87% accurate for four instructions. To ensure moderate (rather than low) load was achieved, four and five swap instructions were used. Items were computer generated and participants were instructed to answer as many questions as they could during the 20-minute drive.

### Procedure

**Figure 2:**
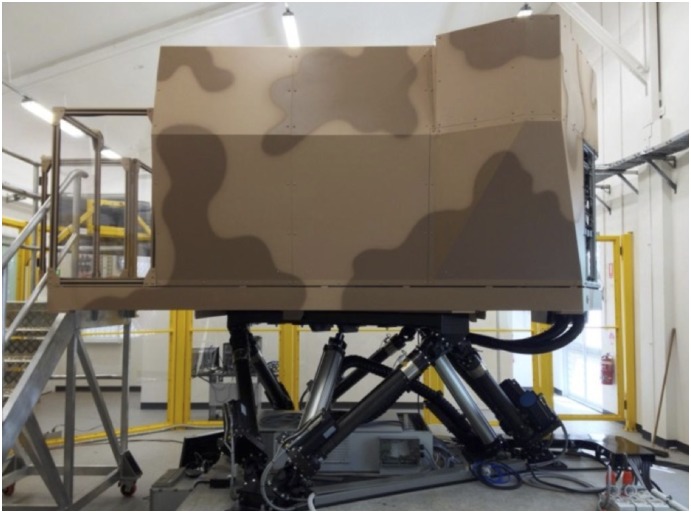
The LAnd Motion Platform (LAMP) simulation facility.

All testing was done with groups of up to four participants per session and conducted in the LAnd Motion Platform (LAMP) simulation facility (see Fig. 2) at the Defence Science and Technology Organisation (DSTO).

Participants were seated inside the 4-seat cabin with motion actuators underneath it. Virtual BattleSpace 2 simulation software (VBS2; [42]) generated windscreen and side windows views through LCD screens inside the cabin, and synchronised them with actuator action in the motion condition of the experiment.

Upon arrival, participants were briefed about the experiment and inducted into the facility. Induction involved assessing participants’ motion sickness propensity with the short motion sickness questionnaire and providing participants with detailed health and safety instructions regarding the LAMP. This included how to enter the LAMP; how to be seated and buckle belts properly; how to stop the simulator at any time if, for example, sickness or discomfort occurred; how to exit in case of an emergency; other relevant details and address any queries the participants may have had.

Once seated in the LAMP cabin, participants were given an iPad to complete all control and evaluative tasks and an attention-switching booklet. They first completed the driving and military experience, and self-control scale questionnaires before completing the first test block: including Medical Decision-Making Test (MDMT), NASA-TLX, and Stroop Test (ST) as described above and in this order. All participants had to complete this test block before the group could proceed. To avoid further lapses in load or arousal for speedier individuals, participants were instructed to work through the attention-switching booklet until everyone was ready to proceed. Once all participants had finished, they remained seated for the 20-minute simulated drive, which was programmed with VBS2 [42]. Driving speed was kept constant and the only sound was of the car engine. Participants then completed the second test block, followed by the second 20-minute drive, and then the third and final test block.

#### Cognitive load

Cognitive load was manipulated within subjects. All participants performed one low-load task of simply sustaining attention on the road as instructions indicated that questions would be asked about what was seen. The other drive involved dual tasking and hence moderate load: sustaining attention on the road and completing the Letter swaps task on the iPad. For this moderate-load drive, the Letter swaps task was the primary task. Attending to the road was secondary but the instruction stressed that questions would still be asked about what was seen. As a manipulation check, participants were asked to report the lowest speed limit they saw immediately after each drive and before the next test block. Overall, participants answered a mean of 45 letter-swap items and 37 correct. Neither the number answered (*t*_67_ = .23, *p* = .82) nor number correct (*t*_67_ = .46, *p* = .65) differed significantly between the low-load-start and moderate-load-start groups.

#### Sustained effect

**Figure 3:**
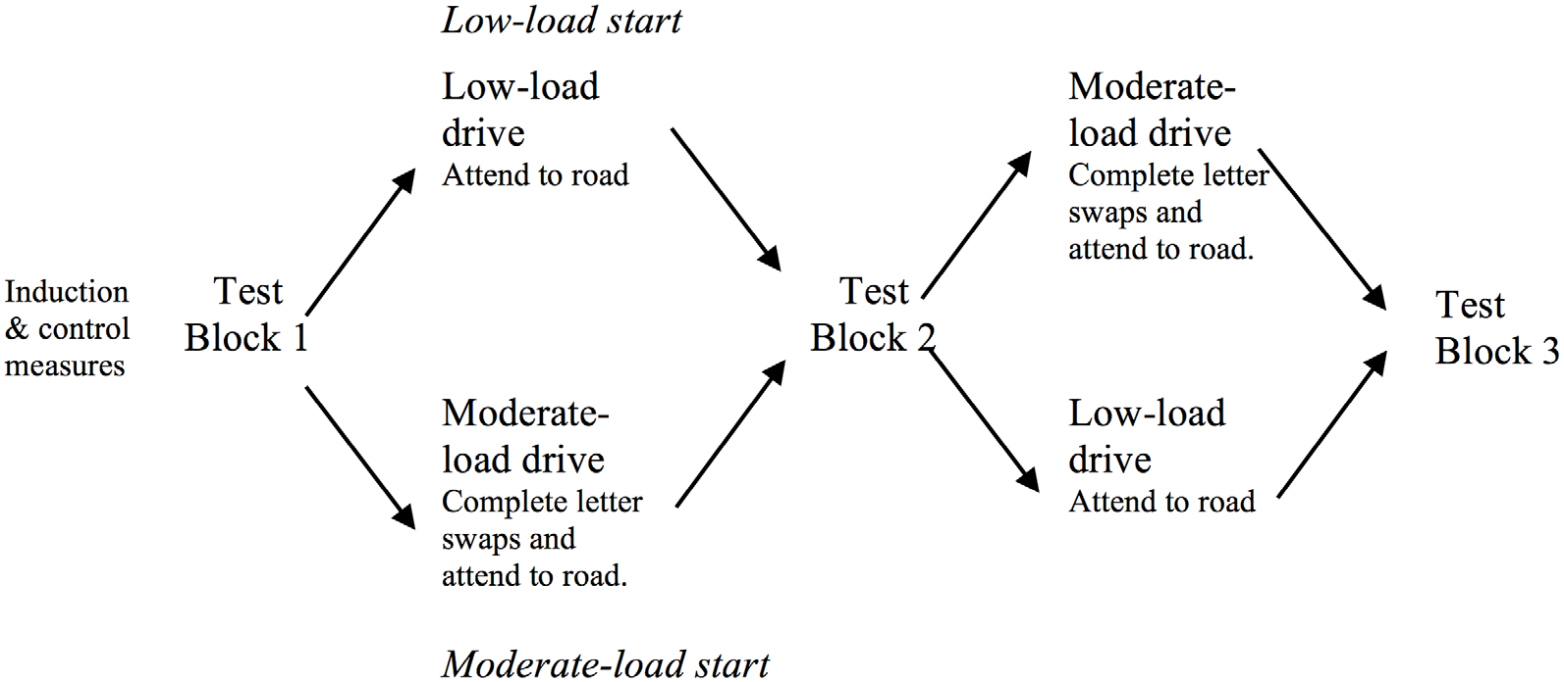
Outline of experimental design for attentional load conditions. The low-load-start group followed the upper sequence; the moderate-load-start group followed the lower sequence.

The potentially sustaining effect of low cognitive load was manipulated between subjects. Ten groups (36 participants) started with the low-load condition in the first drive (between measurement Blocks 1 and 2) and the moderate load in the second drive (between Blocks 2 and 3). This sequence was reversed for the remaining 34 participants, who started with the moderate-load drive first (between Blocks 1 and 2) followed by the low-load drive (between Blocks 2 and 3). These two groups will be respectively referred to as low-load-start and moderate-load-start groups. Should low-load have a sustained effect, the hypothesised improvements following the moderate-load drive should be poorer for the low-load-start group (H3). Fig. 3 outlines the experimental procedure for participants assigned to the low-load-start and moderate-load-start conditions.

#### Arousal

Arousal was manipulated as a between subjects factor. Thirty-seven participants were block-randomised (block size N = 4) to the motion simulation group. For this group, visual simulation was synchronised with motion applied to the entire LAMP cabin (see Fig. 1), with vibration levels averaging around Motion Level 2, which corresponds to a well-maintained unsealed road. The remaining 33 participants performed the same simulated drives with no motion. A counterbalanced design was prepared prior to the two weeks in which testing took place. The final number of participants in each experimental condition is shown in Table 1 below.

**Table 1:** Number of participants in each between-subjects condition.

	Low-load start	Moderate-load start
No Motion	*N* = 17	*N* = 16
Motion	*N* = 19	*N* = 18

## Results

### Descriptive Statistics: evidence of PEs

**Figure 4:**
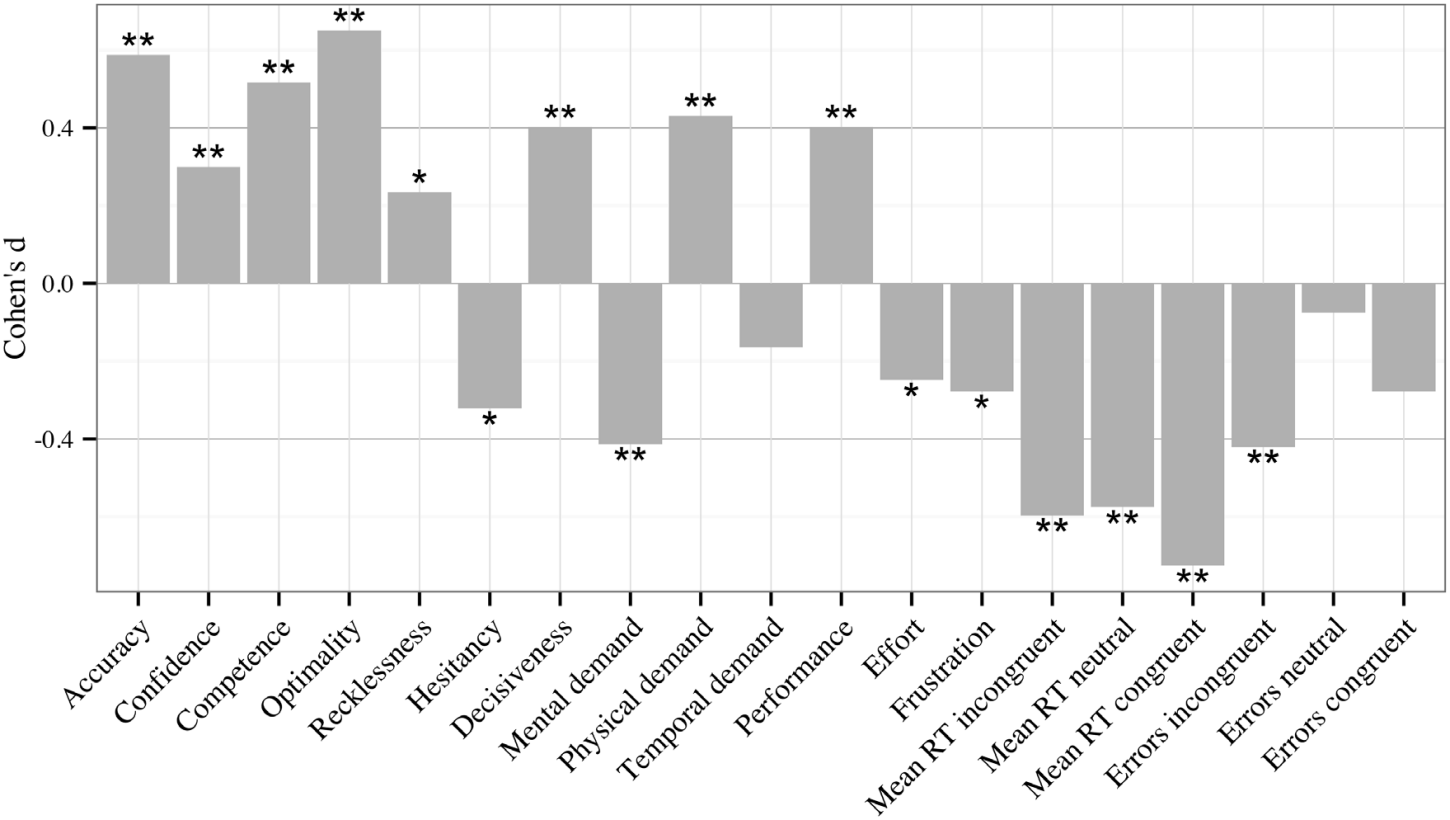
Effect sizes, calculated as Cohen’s d, of the difference between Blocks 1 and 3. Positive effect size represents an increase from Block 1 to 3. Accuracy = MDMT diagnostic accuracy; Confidence = MDMT diagnostic confidence; Competence to Decisiveness = MDMT decision patterns described in introduction and method; Mental demand to Frustration = NASA-TLX ratings described in method; Mean RT = mean reaction time on Stroop test items (incongruent, neutral or congruent items); Errors = error frequency on Stroop test items (incongruent, neutral or congruent items). **p<.01; *p<.05.

Table 2 shows the descriptive statistics and internal consistency estimates for each variable used in the present study across the three testing blocks. Internal consistency estimates were calculated as Cronbach’s alpha where possible, or on the basis of an odd/even item split correct with the Spearman-Brown formula [43], [44]. Additionally, Fig. 4 shows the effect size of the change from Block 1 to 3, and whether the change was statistically significant in a repeated measure ANOVA.

**Table 2:** Descriptive statistics for all variables across three test blocks.

				Block 1								Block 2									Block 3								
		Unit		Mean		SD			IC			Mean			SD			IC			Mean				SD				IC
**MDMT**																													
Accuracy	%		44.57		21.38			0.71			54.55			25.27			0.81			58.51		25.90				0.83			
Confidence	%		56.20		24.21			0.97			61.66			29.47			0.98			64.23		29.13				0.98			
Competence	%		59.15		16.38			0.53			65.71			20.07			0.74			68.30		18.94				0.72			
Optimality	%		34.24		21.77			0.74			45.18			30.77			0.87			51.18		29.71				0.90			
Recklessness	%		57.65		30.35			0.68			60.71			31.23			0.76			65.21		34.11				0.80			
Hesitance	%		26.15		27.42			0.76			26.36			32.48			0.80			17.68		25.25				0.80			
Decisiveness	%		64.76		26.23			0.85			70.09			28.57			0.90			75.54		27.41				0.90			
**NASA-TLX**																													
Mental demand		1–21		15.30		3.40	NA			13.61			4.37			NA			13.65				4.51				NA		
Physical demand		1–21		2.01		1.38	NA			2.84			2.33			NA			3.06				3.13				NA		
Temporal demand		1–21		9.42		5.00	NA			8.84			4.75			NA			8.62				4.69				NA		
Performance		1–21		7.93		5.09	NA			9.77			5.63			NA			10.13				5.85				NA		
Effort		1–21		12.06		4.57	NA			11.51			4.96			NA			10.86				5.11				NA		
Frustration		1–21		10.86		5.60	NA			9.04			5.59			NA			9.28				5.75				NA		
**Simple Reaction Time**																													
Mean		msec		330.96		43.41	0.95			331.61			43.47			0.94			340.42						45.66				0.93
**Stroop**																													
Mean incongruent		msec		986.21		183.47	0.90			924.43			159.11			0.92			878.05					179.10				0.92	
Mean neutral		msec		862.43		158.28	0.91			792.61			137.38			0.91			778.02					134.27				0.91	
Mean congruent		msec		864.60		173.03	0.91			797.46			141.88			0.92			751.02					138.27				0.92	
Errors incongruent		0+		1.33		1.38	0.60			0.84			1.04			0.48			0.83					0.96				0.47	
Errors neutral		0+		0.56		0.75	0.32			0.73			0.82			0.19			0.50					0.78				0.38	
Errors congruent		0+		0.56		0.83	0.35			0.31			0.55			0.10			0.36					0.59				0.20	

IC = Internal Consistency: Cronbach’s alpha for all variables but the decision patterns, calculated as odd/even item split.

Table 2 and Fig. 4 show, as hypothesised (H1), that participants improved in most variables across the three testing blocks, which is likely to be a result of practice. With the exception of performance in two variables getting worse (MDMT recklessness and NASA-TLX physical demand), absolute values of the effect sizes of practice related improvement ranged from weak (*d* = .07) to relatively strong (*d* = .73), with a mean (*d* = .33) indicative of weak to moderate improvement overall. Specifically, significant positive changes (H1.1) were observed for judgement accuracy, on-task confidence, competence, optimality and decisiveness within the MDMT, and post-task performance evaluations in relation to this test (captured by NASA-TLX). Furthermore, significant declines, indicative of better performance (H1.2), were observed for reaction time on all trials and errors on incongruent trials in the Stroop, hesitancy in the MDMT, and post-task evaluations of mental demand, effort and frustration in relation to this test (as measured by NASA-TLX).

Contrary to H1.2, significant increases were observed for recklessness in the MDMT and the post-task evaluation of physical demand in relation to this test. The significant increase in physical demand may have been the result of a heat wave that occurred during the two testing weeks. To ensure participant safety, testing facility and LAMP cabin temperatures were monitored. When cabin temperatures reached a maximum of 28 degrees, participants were offered water between drives. Given that participants had to remain seated in the LAMP cabin for up to three hours during the experiment, this might explain the increase in physical demand. Nonetheless, although significant, this increase was minor–from 2.01 to 3.06 on a 21-point scale–and no participant commented on this aspect or requested the experiment be stopped.

Non-significant but expected trends were observed in only a few variables: errors on neutral and congruent Stroop trials and temporal demand (NASA-TLX). Neutral Stroop trials do not generate prepotent responses and congruent Stroop trials facilitate correct responding. Hence, there is a floor effect for errors on these trials even in Block 1. This was reflected in the unacceptable internal consistency estimates for the Stroop error variables (.10 to .60), while internal consistency for all other variables were generally in the good to excellent range (.53 to.98). Despite the overall non-significant trend in temporal demand, a significant result emerged in the inferential statistics addressed below.

### Inferential Statistics

To investigate the hypothesis that low cognitive load would impede beneficial PEs (H2.1), for each measure, two pre to post load change scores were calculated – for low load and for moderate load respectively. Then, a (2) X 2 X 2 ANOVA (low vs moderate load X low-load start vs moderate-load start X motion vs no motion) was conducted for each outcome measure. Given that all participants went through low and moderate-load conditions, the low vs moderate load was a within-subject factor. The other two factors were constructed as between-subjects factors by randomly assigning participants to the four groups as seen in Table 1. All analyses were originally conducted with age, gender and self-control as covariates. None of these variables contributed significantly to the results and were omitted from the analyses reported.

### H2.1: Cognitive load

**Figure 5:**
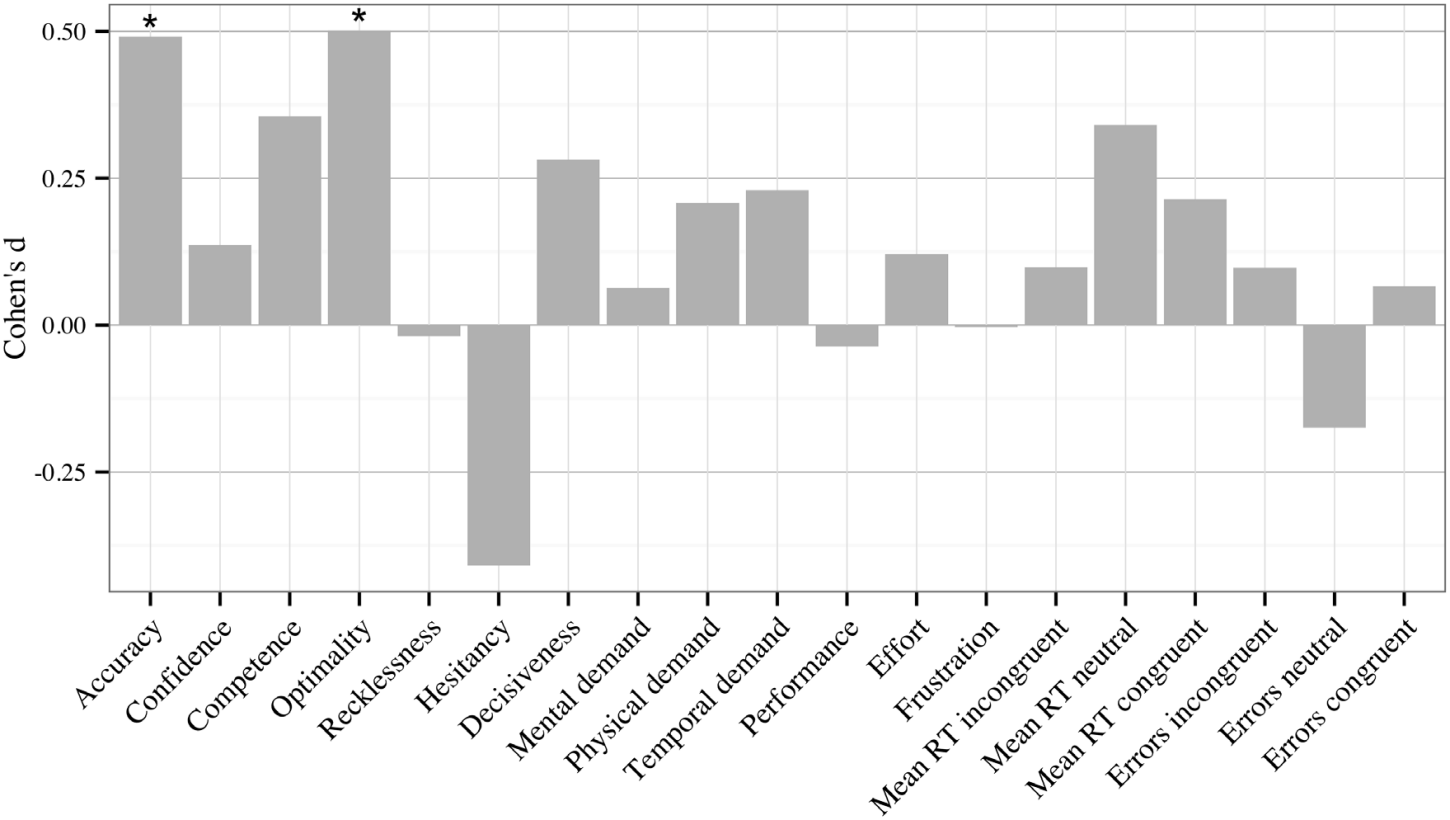
Effect sizes, calculated as Cohen’s d, of the difference between pre to post moderate-load drive change and pre to post low-load drive change. Positive effect size represents a higher pre-post change over the moderate rather than low-load drive. Accuracy = MDMT diagnostic accuracy; Confidence = MDMT diagnostic confidence; Competence to Decisiveness = MDMT decision patterns described in introduction and method; Mental demand to Frustration = NASA-TLX ratings described in method; Mean RT = mean reaction time on Stroop test items (incongruent, neutral or congruent items); Errors = error frequency on Stroop test items (incongruent, neutral or congruent items). **p<.01; *p<.05.

Fig. 5 shows the effect sizes for the workload main effect comparing the moderate-load drive to the low-load drive. As can be seen in Fig. 5, the hypothesised negative effect of low cognitive load (H2.1) emerged in only half the variables and as significant for only judgement accuracy and optimality. While participants improved overall on average (PEs), significantly greater pre-post improvement in MD accuracy (requiring short-term memory) was observed following the moderate-load drive compared to the low-load drive (*M*_difference_ = 10.60; *F*_1,64_ = 6.50, *p* = .01). The same result was obtained for optimality (*M*_difference_ = 10.58; *F*_1,64_ = 6.36, *p* = .01), which is unsurprising given that optimality requires a high degree of accuracy and is most strongly predicted by accuracy measures [21], [22]. Post-hoc analysis revealed the pre to post increase in these variables was significantly greater than zero following the moderate-load drive (*Accuracy*_change_ = 12.59, 95% CI [7.70,17.47]; *Optimality*_change_ = 14.31, 95% CI [8.98,19.65]), but not the low-load drive (*Accuracy*_change_ = 1.99, 95% CI [−3.13,7.12]; *Optimality*_change_ = 3.46, 95% CI [−1.56,8.47]). That is, low cognitive load did not reduce positive PEs on short-term memory accuracy: it completely negated them.

### H2.2: Motion

Motion did not significantly alter any variable. This suggests either that short-term changes in arousal do not impact decision-making processes during practice or that the motion manipulation was not strong enough to obtain an effect. Motion did, however, act as a moderator, which will be discussed in the interaction section below.

### Hypothesis 3: Sustained effect of low cognitive load (order)

**Figure 6:**
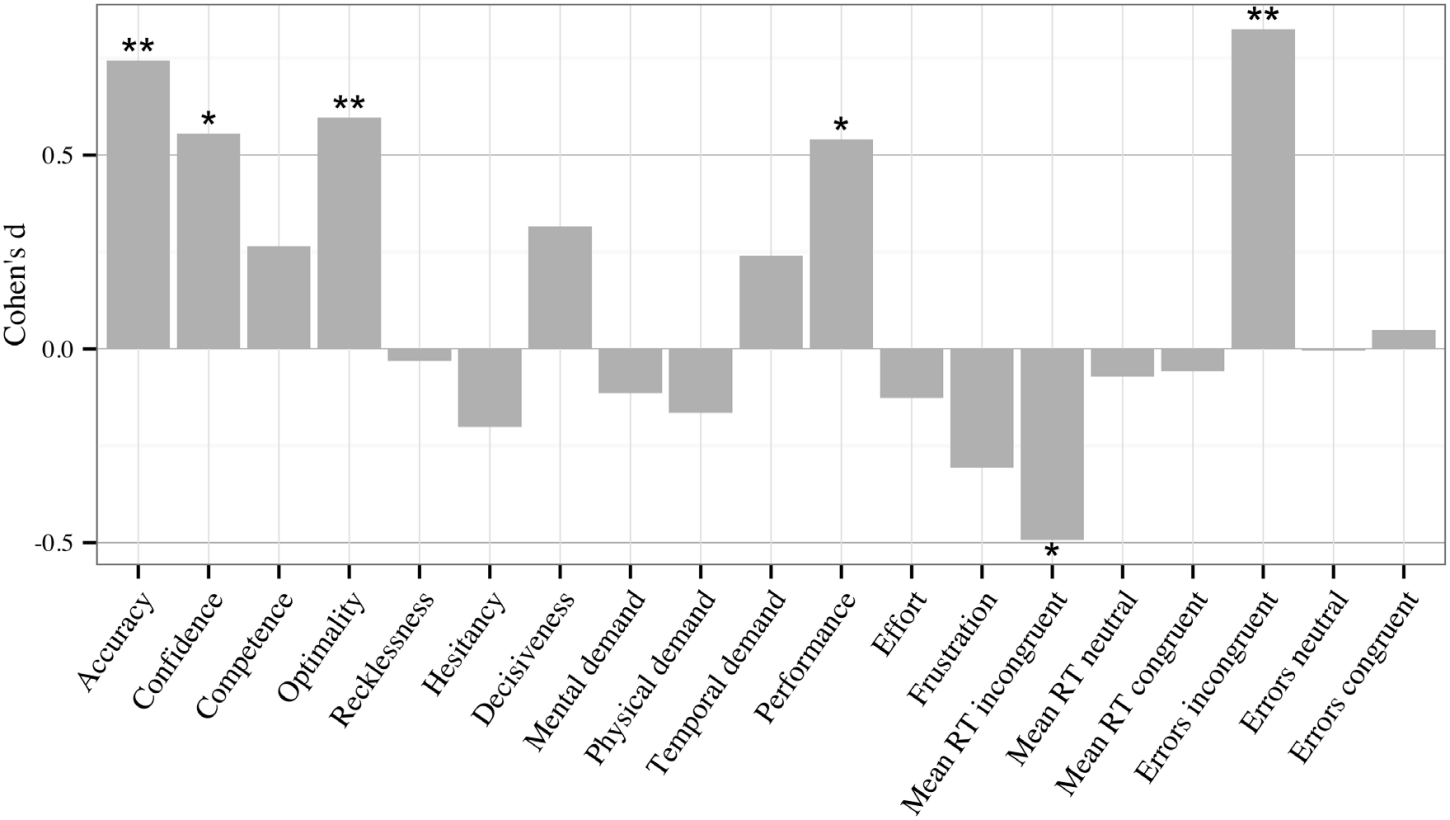
Effect sizes, calculated as Cohen’s d, of the pre to post change difference between low-load-start and moderate-load-start groups. Positive effect size represents a higher score for participants in the moderate-load-start group than the low-load-start group. Accuracy = MDMT diagnostic accuracy; Confidence = MDMT diagnostic confidence; Competence to Decisiveness = MDMT decision patterns described in introduction and method; Mental demand to Frustration = NASA-TLX ratings described in method; Mean RT = mean reaction time on Stroop test items (incongruent, neutral or congruent items); Errors = error frequency on Stroop test items (incongruent, neutral or congruent items). **p<.01; *p<.05.

Fig. 6 shows the effect sizes for the order main effect comparing the moderate-load-start group to the low-load-start group. As hypothesised (H3), the moderate-load-start group had greater overall practice related improvements in all but three variables than the low-load-start group, and five of these expected relationships were significant. Of the expected results, absolute values of the effect sizes ranged from weak (*d*<.01) to strong (*d* = .74), with a mean (*d* = .29) indicative of weak to moderate overall advantage for the moderate-load-start group. Moderate-load-start participants showed significantly greater improvement in reaction time (RT) to incongruent Stroop trials, short-term memory accuracy, optimality and the metacognitive monitoring variables: on-task metacognitive confidence and post-task evaluations of MDMT performance. Despite the expected finding on the Stroop reaction time variable (*M*_difference_ = 32.16; *F*_1,62_ = 4.30, *p* = .04), contrary to the hypothesis, low-load-start participants made significantly fewer errors on incongruent Stroop trials than moderate-load-start participants (*M*_difference_ = −.60; *F*_1,66_ = 11.60, *p*<.01). Post-hoc analysis of these results revealed that both groups significantly improved their reaction times, but only the low-load-start group significantly improved their error rate (*incongruent error*_change_ = −.54, 95% CI [−.78, −.28]).

Similar to the cognitive load results, overall, low-load-start participants showed significantly poorer pre-post improvements in short-term memory accuracy (*M*_difference_ = −9.93; *F*_1,64_ = 10.99, *p*<.01) and optimality (*M*_difference_ = −8.71; *F*_1,64_ = 9.13, *p*<.01). Furthermore, post-hoc analysis of these results revealed that the overall pre-post increase in these variables was significantly greater than zero for moderate-load-start participants (*Accuracy*_change_ = 11.92, 95% CI [7.86,15.98]; *Optimality*_change_ = 13.24, 95% CI [9.05,17.42]), but not significant (accuracy) or only marginally significant (optimality) for low-load-start participants (*Accuracy*_change_ = 2.66, 95% CI [−1.17,6.48]; *Optimality*_change_ = 4.53, 95% CI [.59,8.48]). These results suggest that low-load impeded PEs not only immediately, but also sustained its degrading effect after the workload increased to a moderate level.

Although low cognitive load did not have a significant main effect on the metacognitive monitoring variables, the order in which low-load was experienced did. This suggests that the delayed/enduring effect of low load may be stronger than its immediate effects. Specifically, low-load-start participants showed significantly poorer overall pre-post gains than moderate-load-start participants in on-task metacognitive confidence (*M*_difference_ = −5.36; *F*_1,64_ = 5.94, *p* = .02) and post-task evaluations of MDMT performance (*M*_difference_ = −1.12; *F*_1,64_ = 4.24, *p* = .04). Again, post-hoc analysis of these results revealed that the overall pre-post increase in these variables was significantly greater than zero for moderate-load-start participants (*Confidence*_change_ = 6.83, 95% CI [3.63,10.02]; *Performance*_change_ = 1.64, 95% CI [.84,2.43]) but not low-load-start participants (*Confidence*_change_ = 1.47, 95% CI [−1.55,4.48]; *Performance*_change_ = .52, 95% CI [−.23,1.26]).

### Interactions

**Figure 7:**
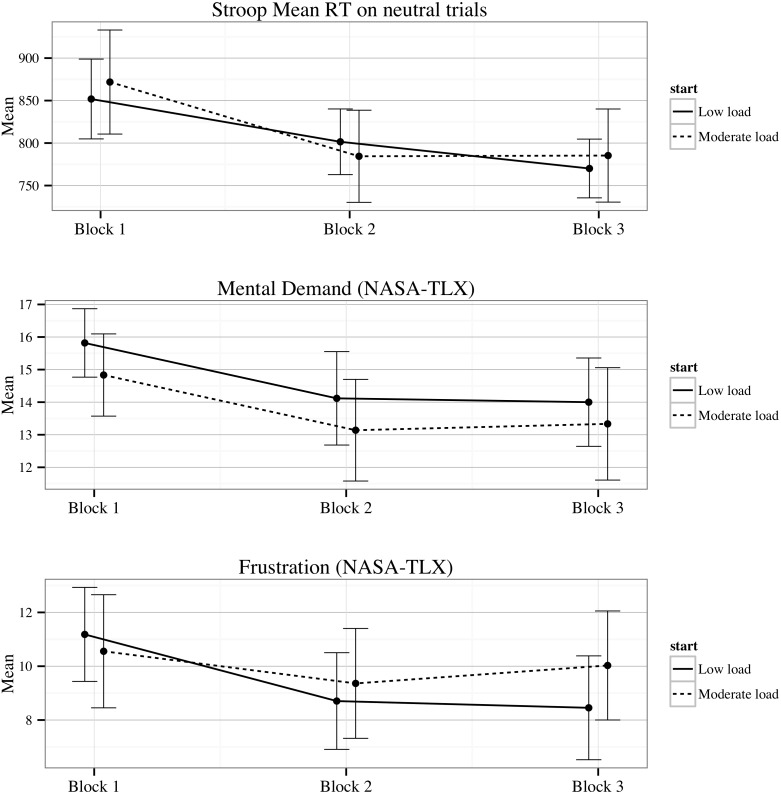
Means and 95% Confidence intervals for Stroop reaction time on neutral trials, and NASA-TLX measures of frustration and mental demand by load condition across the three testing blocks.

Although explicit interaction hypotheses were not formulated, the present research offered the opportunity to examine them. No significant cognitive load by motion interactions emerged. However, three significant load by order interactions emerged for the following: mean reaction time to Stroop neutral trials (*F*_1,62_ = 7.26, *p*<.01); post-task evaluations of MDMT mental demand (*F*_1,64_ = 8.16, *p*<.01); and post-task evaluations of MDMT frustration (*F*_1,64_ = 4.36, *p* = .04). Analysis of these revealed the same pattern of results for all three variables: regardless of the group, participants showed greater pre-post improvement on these variables after their first drive than second drive. This can be seen in Fig. 7 by significant declines in most variables, for both groups, from Block 1 to 2, but not Block 2 to 3.

Two significant results emerged as a result of a motion by order interaction: recklessness (*F*_1,57_ = 7.96, *p*<.01) and post-task evaluations of temporal demand (*F*_1,64_ = 8.13, *p*<.01). These results revealed that the increase in pre-post recklessness for low-load-start participants in the no-motion rather than motion condition (*M*_difference_ = 14.66) was significantly different to the increase in moderate-load-start participants with motion rather than no motion (*M*_difference_ = −7.09).

**Figure 8:**
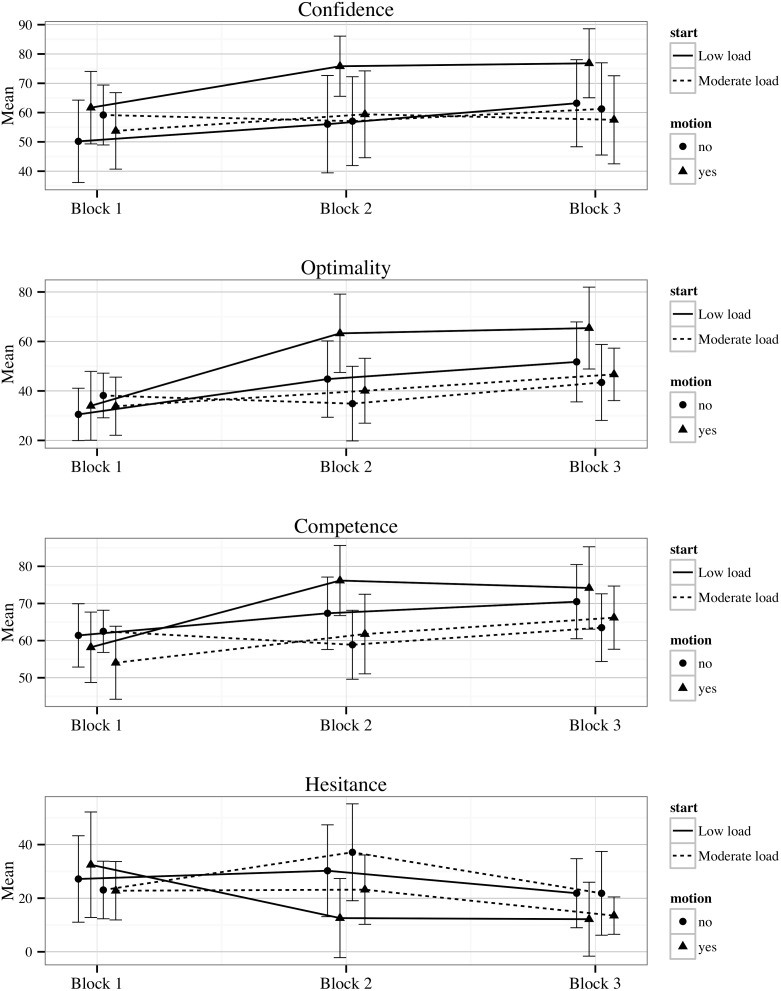
Means and 95% Confidence intervals for MDMT diagnostic confidence, and MDMT decision competence, optimality and hesitance by the between subject conditions (low-load order and motion) across the three test blocks.

Four significant three-way interactions emerged: on-task confidence (*F*_1,64_ = 7.75, *p*<.01), and competence (*F*_1,64_ = 4.35, *p* = .04), optimality (*F*_1,64_ = 4.39, *p* = .04), and hesitancy (*F*_1,64_ = 4.63, *p* = .04). Post-hoc examination of these results revealed that participants showed a significant pre to post drive improvement on all four variables (indicated by a decrease for hesitancy) given only one condition: when both moderate workload and motion were experienced on the first drive. This can be seen by the significant change from Block 1 to Block 2 on these variables for the moderate-load-start group who also had motion only (dotted line; triangle markers) in Fig. 8.

## Discussion

The present research investigated how low cognitive load and the absence of arousal affected typical practice effects on cognitive and decision-making tasks. The potential for optimal performance is known to occur when levels of cognitive load and arousal (experienced through physical stimulation such as motion when driving) are neither too high nor too low. Low levels of either (the present focus) can lead to the mind slowing and generating distractions. For example, student learning is inhibited when material is too easy [45], and undemanding and under-arousing monotonous highways are associated with greater frequencies of accidents (see [14]). Similarly, people tend to create work for themselves, often irrelevant to the task at hand, when the amount of information they are required to process is much less than the amount they are capable of processing [3], [5], and sustaining low cognitive load has enduring negative effects that inhibit vigilance performance [4]. However, the effects of low cognitive load and the absence of arousal on practice-related improvements (Practice Effects [PEs]) have not been investigated. The present study therefore assessed simultaneous changes in inhibitory control, short-term memory, metacognitive monitoring, and decision competence over repeated testing blocks (allowing for practice) under conditions of low or moderate cognitive load and motion or no-motion. Overall, low cognitive load impeded PEs on most variables, and either low cognitive load or the absence of motion was sufficient to impede a subset, suggesting two distinct pathways that may cause a decline in cognitive performance (e.g. [18]).

### Inhibitory control

Inhibitory control, an executive function required to override the prepotent Type 1 processes to analytic and effortful Type 2 thinking processes, was assessed with the Stroop colour-naming test. As hypothesised, participants improved their reaction time on all trial types (congruent, neutral and incongruent) as a result of practice. This is most likely linked to the way in which learning occurs on the Stroop task. In addition to inhibitory control, working memory is involved, as participants must remember which button corresponds to which colour to respond quickly and correctly [24]. The Stroop PEs therefore best reflected procedural learning via an overall improvement in internalising the instruction. That is, participants likely internalised where each of the response options was located in order to respond faster. For future studies, alternative inhibitory control tasks that place less demand on working memory (e.g., spatial Stroop) may help confirm this.

Overall, the results suggest that low cognitive load impeded, but did not eliminate, an overall improvement of Stroop task performance despite a return to moderate load. As hypothesised, moderate-load-start participants became faster on incongruent Stroop trials than low-load-start participants. That is, moderate-load-start participants specifically improved their response time on trials requiring the inhibition of prepotent response alternatives compared to low-load-start participants. Low-load-start participants, however, significantly decreased their incongruent error rate whereas moderate-load-start group did not. This may have been the result of a speed-accuracy trade off, but this is difficult to confirm. That is, moderate-load-start participants may have internalised this response mapping better enabling them to respond faster, but also making them more susceptible to errors. Keeping this possible trade-off in mind, the present results do suggest that low load may reduce improvements in inhibitory control on practiced tasks. This indicates that moderate cognitive load may be required for task specific inhibitory control improvements.

### Short-term memory

Short-term memory ability was assessed via diagnostic accuracy in the MDMT as participants were required to memorise symptoms and their associated diseases to make accurate diagnoses. The symptom list and diseases were intentionally altered across blocks so that procedural (strategy), but not declarative (content) learning could occur. Strong and significant PEs emerged for this variable, demonstrating that participants were able to refine their memory strategies in this task and improve their judgement accuracy successfully with practice.

Low cognitive load appeared to eliminate these positive PEs. The effect of low load on these PEs was so detrimental that it occurred in both an immediate and enduring capacity. That is, participants showed no significant signs of improving their judgement accuracy once they had experienced the low-load task (following both the low and moderate-load drive). This suggests that operators may be unable to refine cognitive strategies with practice on critical tasking amidst prolonged periods of low cognitive load (e.g., monitoring autonomous vehicles), even after the low-load is replaced with a more engaging task. These results therefore suggest that maintaining moderate cognitive load by processing roughly as much information as one is able to is important for PE to occur and for cognitive strategies (at least involving short-term memory) to improve.

### Monitoring

Mimicking the above results, the monitoring variables demonstrated typical PEs [33] such that participants felt more confident in their judgements and found the MDMT become easier overall with each test block. Specifically, on- and post-task metacognitive assessments of accuracy increased, and post-task assessments of mental demand and frustration decreased over the course of the experiment. Also in line with the accuracy results, these practice-related changes only emerged for participants who began with the moderate-load drive, but no such pattern was observed for those who began with the low-load drive. That is, participants’ overall metacognitive assessments of performance aligned with the overall changes in accuracy as a result of practice.

Unlike their accuracy results, participants did not show an increase in confidence specifically following the moderate-load drive. The only significant pre to post change in on-task confidence was for participants who experienced moderate workload and arousal (motion) on their first drive. That is, while metacognitive monitoring reflected performance overall, it did not appear to be sensitive to changes within the experiment for participants who had experienced low cognitive load or no motion. These results suggest that low levels of cognitive load or arousal may lead to monitoring and decision errors.

The dissociative change of accuracy and metacognitive monitoring is an important concern. Monitoring experiences, not judgement accuracy, guide decisions [31], [46]. Operators are only capable of making competent decisions if they hold inaccurate information about the world when their monitoring system detects their inaccuracy. If accuracy and monitoring are dissociated, however, this may lead to reckless or hesitant decision errors. For example, operators may more accurately assess situations with practice but not necessarily detect these improvements if they have been learning under conditions of low cognitive load or arousal. They are therefore unlikely to change their decision making and capitalise on their improved assessments. Similarly, under-loaded students may think they are not improving when they in fact are, in turn contributing to their lack of motivation and desire to avoid further learning [7]. The implication is an important one: people may not be able to detect important changes in their accuracy following periods of low cognitive load or arousal.

### Decision competence

As hypothesised, the decision variables indicated that participants tended to make better decisions with practice. That is, competence, optimality and decisiveness within the MDMT increased, while hesitancy decreased. Unexpectedly, however, recklessness also increased. Discussed below, these results suggest that the increase in accuracy and confidence drove changes in decisiveness overall rather than individuals refining their decision making competence.

In line with the on-task confidence results, pre to post decision behaviour improved only in those participants who experienced moderate workload and motion on their first drive. Here, participants significantly increased their competence and optimality, as well as decreased their hesitancy. These variables are linked through behaviour following accurate judgements. That is, greater confidence in correct judgements would lead to an increase in hits (primary-goal-related decisions following accurate judgements) and decrease in misses (ancillary-goal-related decisions following accurate judgements). This would in turn increase optimality (overall percentage of hits) and competence (overall percentage of hits and correct rejections), albeit to a lesser extent than optimality, and a decrease in hesitancy (percentage of misses following accurate judgements). That is, participants were able to significantly improve their judgement accuracy and to capitalise on it if they received both moderate cognitive load and motion. Conversely, either low load or no motion impeded improvements in these variables. Hence, being unable to detect improvements in one’s assessments as a result of low load or arousal was associated with being unable to translate those improvements into better decision making.

An unexpected finding was that recklessness increased, demonstrating a decrease in decision performance following inaccurate judgements. That is, although practice decreased the overall frequency of inaccurate assessments, the decisions following them became worse. Taking the other results into consideration, this suggests that participants became more confident and more decisive in general without discriminating between correct or incorrect judgements. The finding that competence increased only half as much as optimality lends further support to this. Participants therefore managed to increase their proportion of hits as a result of increased accuracy and confidence, but not their correct rejections. Overall, the results suggest that along with accuracy and confidence, practice may improve decisiveness and optimality, but it is unlikely to improve overall competence and the capacity to discriminate correct from incorrect decisions.

### Implications

There are a number of key implications of the present study for fatigue as a result of low load and arousal, practice effects and their interactions. First, low cognitive load appeared to have the greatest detrimental effect overall. It impeded PEs immediately following the manipulation and even after a period of moderate-load tasking. This is an important finding for operator performance, which often follows prolonged periods of low load. For example, it is crucial that unmanned vehicle operators be able to benefit from practice during infrequent but essential tasking periods. The minor degree of cognitive load they typically experience, however, may inhibit PEs and the refinement of task relevant strategies from taking place. Training that includes a high frequency of intense tasking, thus avoiding low mental load, should be utilised to ensure improvement occurs in these contexts.

The present study suggests that operator strategies designed to mitigate suboptimal learning as a result of task disengagement may need to take cognitive load and arousal into consideration, as well as the factors they are targeting. For example, sustaining cognitive activity alone may maintain PEs related to the improvement of short-term memory, but may not support accurate monitoring. Both cognitive load and arousal should be maintained, with procedures developed that mitigate their decline.

A further key implication is that practice does not appear to alter the successful detection of incorrect decisions. That is, even skilled and well-practiced individuals may be unlikely to detect incorrect decisions: an important implication in high-risk contexts in which incorrect judgements are frequent (e.g., see [47]). For example, experienced commanders may hold unwarranted confidence in new environments thus immediately and incompetently taking dangerous routes. Where appropriate, the application of procedures designed to increase awareness of these tendencies under conditions of low load and arousal is important for priming more cautious decision-making processes in novel and high-risk contexts, particularly for practiced and confident individuals.

### Limitations and recommendations

The results of the present study are limited in a number of ways that will be important to address in future research. First, participants’ cognitive resources may have become depleted (overloaded) as a result of sustaining task load for up to three hours. That is, the results–of the third block in particular–may reflect a depletion (rather than disengagement) of cognitive resources in addition to the manipulations. This might explain the ‘sustaining’ effects simply as overload impeding performance in the final block. The NASA-TLX ratings of mental and temporal demand did not increase as would have been expected if depletion occurred, however [3]. Future research should aim to isolate the effects of cognitive resource depletion, e.g. via sustained overload or sleep deprivation, on the variables studied here.

A further concern may be the strength and specificity of the manipulations. For example, the motion manipulation played a minor role in the results. Despite being more arousing than no motion, it may be that the degree of motion used was not enough to be classed as ‘moderate’. The motion level used here was chosen because of motion sickness concerns. However, no participant reported feeling unwell suggesting that an increase in motion is warranted in future studies. Furthermore, the low-load drive appeared to have an overwhelmingly large effect. It may be that this drive was somewhat under stimulating in addition to imposing low cognitive load. Although the road was designed to avoid it, this too is difficult to confirm. We feel confident that the motion manipulation can be increased in future research and recommend that measures be employed to tease apart the arousal/load demands (e.g., via self-reported ratings) in future research.

Finally, it will be important in the future to investigate how low cognitive load and arousal contribute to task disengagement. The present research was focussed on these variables due to their shared link across boredom and passive fatigue literature, which both infer that these variables result in task disengagement. While the results of the present study, showing that these variables impede practice effects, aligns with literature in these fields, it is not possible to confirm whether the cause was task disengagement. Follow up studies will benefit from attempting to measure levels of task engagement in order to confirm this hypothesis laid out in the boredom and passive fatigue literatures.

## Supporting Information

S1 Data (ZIP)
