# Correction: Low Cognitive Load and Reduced Arousal Impede Practice Effects on Executive Functioning, Metacognitive Confidence and Decision Making

**DOI:** 10.1371/journal.pone.0119113

**Published:** 2015-03-23

**Authors:** The PLOS ONE Staff

Figs. 7 and 8 are incorrect. The authors have provided the correct versions here.

**Fig 7:**
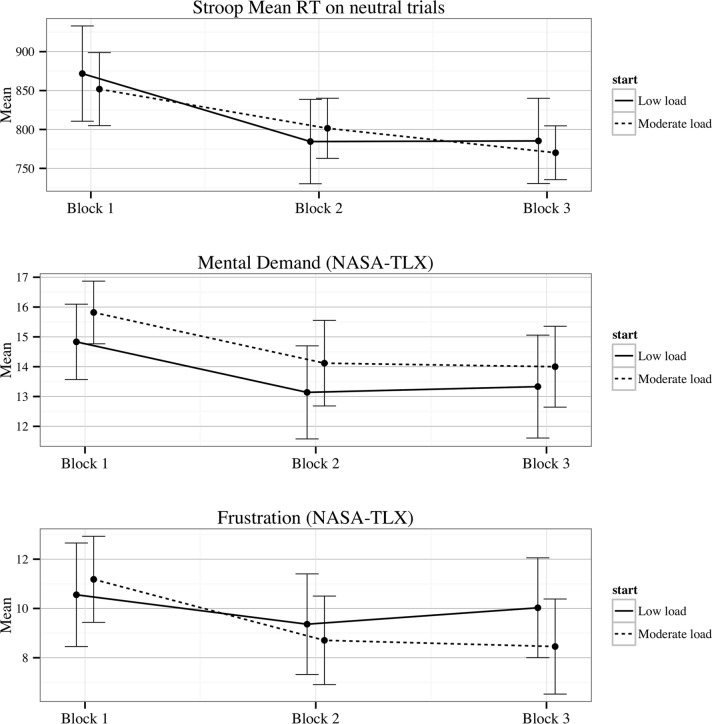
Means and 95% Confidence intervals for Stroop reaction time on neutral trials, and NASA-TLX measures of frustration and mental demand by load condition across the three testing blocks.

**Fig 8:**
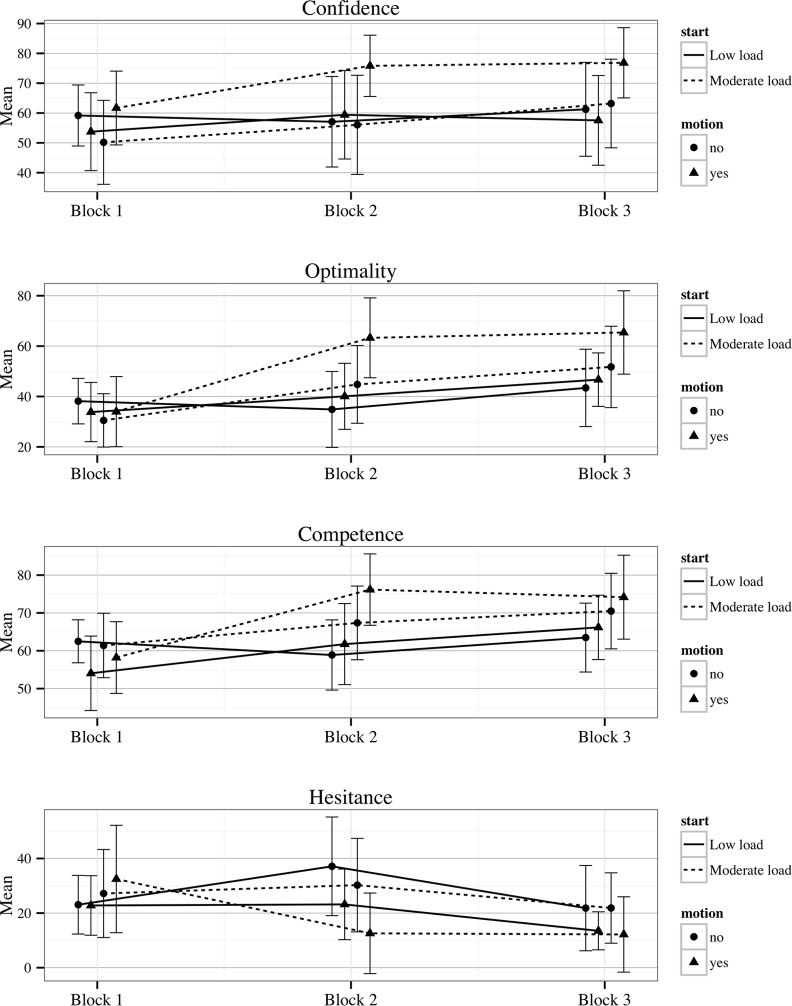
Means and 95% Confidence intervals for MDMT diagnostic confidence, and MDMT decision competence, optimality and hesitance by the between subject conditions (low-load order and motion) across the three test blocks.
